# Quantifying the Length of Stay and Economic Impact of Albuterol and Levalbuterol in Hospitalized Patients With Chronic Obstructive Pulmonary Disease: A Retrospective Cohort Study

**DOI:** 10.7759/cureus.59039

**Published:** 2024-04-26

**Authors:** Xun Liu, Hongmei Zhang, Zaixing Yang, Yalan Ran, Yao Qiu, Li Wang, Liang Zeng, Xuan Li, Canghong Zhi, Junyu Lu

**Affiliations:** 1 Department of Pulmonary and Critical Care Medicine, The People’s Hospital of Yongchuan District, Chongqing, CHN; 2 Department of Pulmonary and Critical Care Medicine, The Fifth People’s Hospital of Chongqing, Chongqing, CHN; 3 Department of Statistics, NanPeng Artificial Intelligence Research Institute, Chongqing, CHN; 4 Department of Medicine, Joincare Pharmaceutical Group Industry Co. Ltd., Shenzhen, CHN

**Keywords:** albuterol, levalbuterol, direct costs, hospitalization, chronic obstructive pulmonary disease

## Abstract

Introduction

Chronic obstructive pulmonary disease (COPD) affects millions in China and imposes a considerable economic burden on hospitalized patients who experience exacerbations. Nebulized short-acting beta-2 agonists (SABA) are recommended as initial therapy for exacerbation patients, but the optimal SABA remains uncertain. This study aimed to evaluate the impact of different SABAs, such as albuterol and levalbuterol, on the length of stay (LOS) and direct medical costs among hospitalized patients diagnosed with COPD.

Methods

This retrospective cohort study uses linked hospital administrative data from three hospitals in Chongqing. Patients with COPD, aged 40 years and older, who had been continuously treated with nebulized albuterol or levalbuterol during hospitalization, were eligible for the study. Patients were matched 1:1 by sex, age, and severity according to the Global Initiative for Chronic Obstructive Lung Disease (GOLD) grades 1-4. Patients were grouped according to the different SABA treatments they received. Demographic, economic, and clinical data were retrieved. LOS and direct healthcare costs were assessed.

Results

A total of 158 COPD patients were included, with 79 in each treatment group. Patients treated with levalbuterol had a significantly shorter median LOS (7.0 days vs. 8.0 days, P=0.003) and fewer direct healthcare median costs (total cost: ¥8,868.3 vs. ¥10,290.7, P=0.014; COPD-related western medicine fees: ¥383.8 vs. ¥505.3). Patients aged 60 or older were more likely to experience longer LOS and higher direct costs.

Conclusion

This retrospective cohort analysis supports that albuterol was associated with longer LOS and higher costs than levalbuterol.

## Introduction

Chronic obstructive pulmonary disease (COPD) affects about 99.9 million people in China [1] and is a considerable economic burden because of exacerbations that can lead to hospitalization or emergency visits [2]. In China, hospitalization represents over 75% of all COPD-related healthcare costs [3], and almost 29.0% of COPD patients experience two or more hospitalizations each year [4]. Hospital-related costs are estimated at approximately ¥20,118/year per patient [5] but costs increase with the frequency and severity of exacerbation.

Nebulized short-acting beta-2 agonist (SABA) treatment, such as albuterol [6], is recommended as the initial therapy for acute exacerbation of COPD (AECOPD) in guidelines [7,8]. Levalbuterol, the R-isomer of albuterol and a newer SABA treatment [9] has been suggested to reduce the one-day length of stay (LOS), with $554 direct hospital cost savings per COPD patient [10]. However, this is not a universal pattern [11-13]. Conflicting results have made levalbuterol’s benefits in LOS and direct hospital costs ambiguous, and there was no validated evidence among the Chinese population. To improve patient outcomes and reduce the healthcare system burden, there is a need to determine the optimal SABA treatment for AECOPD in China. This study aims to investigate whether using levalbuterol in hospitalized COPD patients in Chongqing reduces the LOS and associated medical costs as no previous studies have been conducted in China on this topic.

## Materials and methods

Study design, data source, and period

Based on data collected from June 2021 to July 2022, a retrospective cohort analysis was performed at three hospitals in Chongqing, China. The study utilized a computerized medical records database to capture patient information, including demographic data, clinical diagnoses, administrative records (including admission and discharge dates and methods), comorbidities, and prescribed medications.

This study obtained approval from the medical ethics committee of The Fifth People's Hospital of Chongqing. The approved number is 2022CQSDWRMYYEC-010 (August 8, 2022). Because of the study's retrospective nature, the ethics committee waived the need for informed consent from patients. In compliance with the principles of the Declaration of Helsinki, patient data were anonymized throughout the study.

Study population, inclusion, and exclusion criteria

The study included hospitalized patients diagnosed with COPD meeting the following inclusion criteria: (1) aged ≥40 years; (2) hospitalized patients with a clinical diagnosis of COPD (i.e., the ratio of forced expiratory volume in 1 second (FEV1) to forced vital capacity (FVC) < 0.70) in the previous 12 months [14]; (3) patients who received nebulized levalbuterol or albuterol during the hospital stay. The exclusion criteria comprised (1) patients without complete medical records (e.g., missing demographics, comorbidity, risk factors, medical history, or medication information); (2) patients who changed SABA therapy during hospitalization (e.g., from albuterol to levalbuterol or terbutaline). The two groups were probabilistically matched using sex, year of birth, and COPD severity according to Global Initiative for Chronic Obstructive Lung Disease (GOLD) Grades 1-4 after inclusion and exclusion criteria were applied. The matched sample was stratified in a ratio of 1:1 as the following two cohorts: (1) patients who received nebulized albuterol as an inpatient prescription and (2) patients who received nebulized levalbuterol as an inpatient prescription.

Demographic and clinical variables

Multiple demographic and clinical variables were extracted from the database, including age (40-59 years (younger age); 60-79 years (middle age) or 80-100 years (older age)); sex (male or female); body mass index (calculated from height and weight measurements) [15]; smoking status (current smoker, former smoker, nonsmoker); duration of COPD; lung function (GOLD grade, FEV1/FVC); modified Medical Research Council (mMRC) dyspnea scale (Grades 0-4) [16]; specific comorbidities (e.g., diabetes, gastroesophageal reflux disease, acute myocardial infarction, congestive heart failure, solid tumor, and hypertension); the number of outpatient, inpatient, and ICU visits and emergency department visits in the previous year; medications prescribed for COPD (short-acting bronchodilators (SABD); inhaled corticosteroids (ICS); long-acting beta agonists (LABA); long-acting muscarinic antagonist (LAMA); ICS/LABA; LAMA/LABA; ICS/LAMA/LABA; antibiotics; and glucocorticoids were considered as COPD-related medicine) during hospitalization, duration of nebulized SABA, the LOS, the total direct costs, Western medicine fees, and COPD-related western medicine fees.

Statistical analysis

Categorical variables were presented as numbers and proportions, while normally distributed continuous variables were presented as means and standard deviations (SDs). Non-normally distributed variables were expressed as the median and interquartile range, representing the lower to upper quartile. Categorical variables were analyzed using chi-squared or Fisher’s exact tests, while continuous variables were evaluated using Student’s t-test or Mann-Whitney non-parametric test. Group comparisons were performed using t-tests or Wilcoxon tests.

We employed a simple logistic regression method to investigate the association between independent variables and direct medical costs and LOS. We used a backward stepwise selection procedure to construct a multiple logistic regression model, including only independent variables with a P value of <0.10 in simple logistic regressions. Odds ratios (ORs) and their corresponding 95% confidence intervals (CIs) were calculated. Statistical Analysis System (SAS, version 9.4; SAS Institute Inc., Cary, NC) was used for all statistical analyses. All tests were two-tailed, and significance was determined at a level of P < 0.05.

## Results

Baseline characteristics

Approximately 158 COPD patients, with 79 in each treatment group, met the study's inclusion and exclusion criteria. The mean (±SD) age of COPD patients was 70.0 (±8.9) years, and the majority were male (n = 130; 82.3%). Table 1 presents the baseline summary statistics, which indicate no significant differences in demographic characteristics; GOLD grade; bronchodilators; glucocorticoids; antibiotic therapies; comorbidities; and medical resource usage in the past year between the two groups, except for congestive heart failure (6.3% vs. 16.5%, P = 0.045); emergency visits in the past year (0.2 ± 0.7 vs. 0.7 ± 1.1, P = 0.001); and usage of long-acting bronchodilators (7.6% vs. 29.1%, P < 0.001).

**Table 1:** Baseline characteristics Data are presented as count (%) or mean ± standard deviation or median (Q1-Q3). ^a^Wilcoxon rank sum P value; ^b^Chi-square P value; ^c^Fisher's exact P value; ^d^Two-sample T-test P value BMI: body mass index; mMRC: modified Medical Research Council (mMRC) dyspnea; FEV1: Forced expiratory volume in 1 second; FVC: Forced vital capacity; GOLD, Global Initiative for Chronic Obstructive Lung Disease; SAMA: short-acting muscarinic agonist; ICS: Inhaled corticosteroids; LABA: long-acting beta-agonists; LAMA: short-acting muscarinic agonist; SC: Systemic corticosteroid

	Total (N=158)	Levalbuterol (N=79)	Albuterol (N=79)	P value
Age (years)	70.0±8.9	70.5±9.0	69.5±8.8	0.480^d^
Male, n (%)	130 (82.3%)	66 (83.5%)	64 (81.0%)	0.677^b^
BMI	21.9±3.9	21.8±3.6	22.0±4.1	0.775^d^
Smoking status, n (%)	-	-	-	0.606^b^
Never	45 (28.5%)	24 (30.4%)	21 (26.6%)	-
Current	57 (36.1%)	30 (38.0%)	27 (34.2%)	-
Former	56 (35.4%)	25 (31.6%)	31 (39.2%)	-
Duration of COPD (years)	5.0 (2.0, 10.0)	5.0 (2.0, 10.0)	6.0 (2.0, 10.0)	0.287^a^
mMRC	3.0 (2.0, 3.0)	3.0 (2.0, 3.0)	3.0 (2.0, 3.0)	0.928^a^
mMRC 0, n (%)	0	0	0	-
mMRC 1, n (%)	16 (10.1%)	8 (10.1%)	8 (10.1%)	-
mMRC 2, n (%)	44 (27.8%)	22 (27.8%)	22 (27.8%)	-
mMRC 3, n (%)	79 (50.0%)	40 (50.6%)	39 (49.4%)	-
mMRC 4, n (%)	19 (12.0%)	9 (12.7%)	10 (11.4%)	-
GOLD grade, n (%)	-	-	-	0.066^b^
GOLD 1 (FEV_1_% predicted≥80)	13 (8.2%)	11 (13.9%)	2 (2.5%)	-
GOLD 2 (50≤FEV_1_% predicted<80)	39 (24.7%)	20 (25.3%)	19 (24.1%)	-
GOLD 3 (30≤FEV_1_% predicted<50)	62 (39.2%)	28 (35.4%)	34 (43.0%)	-
GOLD 4 (FEV_1_% predicted<30)	44 (27.8%)	20 (25.3%)	24 (30.4%)	-
FEV_1_/FVC	50.4±9.6	51.0±9.0	50.0±10.2	0.422^d^
Number of comorbidities, n (%)	-	-	-	0.633^b^
0	85 (53.8%)	43 (54.4%)	42 (53.2%)	-
1	51 (32.3%)	27 (34.2%)	24 (30.4%)	-
≥2	22 (13.9%)	9(11.4%)	13 (16.5%)	-
Comorbidities, n (%)	-	-	-	-
Diabetes	18 (11.4%)	8 (10.1%)	10 (12.7%)	0.617^b^
Acute myocardial infarction	4 (2.5%)	3 (3.8%)	1 (1.3%)	0.620^c^
Congestive heart failure	18 (11.4%)	5 (6.3%)	13 (16.5%)	0.045^b^
Hypertension	50 (31.6%)	27 (34.2%)	23 (29.1%)	0.494^b^
Solid tumor	4 (2.5%)	1 (1.3%)	3 (3.8%)	0.620^c^
Gastroesophageal reflux	4 (2.5%)	2 (2.5%)	2 (2.5%)	1.000^c^
Exacerbation within the previous year	-	-	-	-
Emergency visits per patient	0.4±0.9	0.2±0.7	0.7±1.1	0.001^d^
Outpatient visits per patient	3.7±3.2	4.2±3.9	3.3±2.3	0.066^d^
Inpatient visits per patient	1.7±1.1	1.7±1.0	1.7±1.2	0.777^d^
Patients hospitalized in the past year, n (%)	67 (42.4%)	35 (44.3%)	32 (40.5%)	0.629^b^
Other short-acting bronchodilators (yes), n (%)	-	-	-	-
SAMA	6 (3.8%)	2(2.5%)	4 (5.1%)	0.681^c^
Theophylline	158 (97.5%)	75 (94.9%)	79 (100%)	0.120^c^
Long-acting bronchodilators (yes), n (%)	29 (18.4%)	6 (7.6%)	23 (29.1%)	<.001^b^
ICS/LABA	16 (10.1%)	6 (7.6%)	10 (12.7%)	0.292^b^
LABA/LAMA	2 (1.3%)	0	2 (2.5%)	0.497^c^
LAMA	5 (3.2%)	0	5 (6.3%)	0.059^c^
ICS/LABA/LAMA	6 (3.8%)	0	6 (7.6%)	0.028^c^
Glucocorticoids, n (%)	132 (83.5%)	62 (78.5%)	70 (88.6%)	0.086^b^
ICS alone	38 (24.1%)	14 (17.7%)	24 (30.4%)	0.063^b^
SC alone	78 (49.4%)	39 (49.4%)	39 (49.4%)	1.000^b^
ICS+SC	16 (10.1%)	9 (11.4%)	7 (8.9%)	0.598^b^
Antibiotics (yes), n (%)	145 (91.8%)	70 (88.6%)	75 (97.9%)	0.246^c^
1	129 (81.6%)	64 (81.0%)	65 (82.3%)	-
≥2	16 (10.1%)	6 (7.6%)	10 (12.7%)	-

LOS and costs

Regarding the clinical outcomes of interest, the levalbuterol group had a shorter LOS (7.0 (5.0, 9.0) vs. 8.0 (7.0, 10.0), P = 0.003) and nebulized SABA (6.0 (5.0, 7.0) vs. 7.0 (6.0, 9.0), P < 0.001). Furthermore, the albuterol group demonstrated significantly higher median (IQR) of total hospitalization and COPD-related costs compared to the levalbuterol group (total: ¥10,290.7 (7,640.0, 12,542.5) vs. ¥8,868.3 (5,904.4, 11,426.9), P = 0.003; COPD-related: ¥505.3 (348.2, 645.1) vs. ¥383.8 (209.0, 556.8), P = 0.010). The disparity in total cost between the two groups was ¥1,422.4, with a ¥121.5 difference in drug cost. Costs associated with total western medicine fees were not significantly different between patient subgroups (albuterol: ¥2,629.7 (1,457.0, 3,659.1), levalbuterol: ¥2(,720.2 (1,904.6, 4,036.1), P = 0.085). Details are shown in Table 2.

**Table 2:** LOS and costs ^a^Wilcoxon rank sum P value; SABA: short-acting beta-2 agonist

	Levalbuterol	Albuterol	P value
Length of hospital stays	7.0 (5.0, 9.0)	8.0 (7.0, 10.0)	0.003^a^
Length of nebulized SABA	6.0 (5.0, 7.0)	7.0 (6.0, 9.0)	<.001^a^
COPD-related Western medicine fees	383.8 (209.0, 556.8)	505.3 (348.2, 645.1)	0.010^a^
Total Western medicine fee	2,629.7 (1,457.0, 3,659.1)	2,720.2 (1,904.6, 4,036.1)	0.085^a^
Total hospital costs	8,868.3 (5,904.4, 11,426.9)	10,290.7 (7,640.0, 12,542.5)	0.014^a^

Risk factors associated with LOS and costs

Tables 3-4 present the results from univariate and multivariate logistic regression analyses examining the relationship between hospital costs and LOS. Univariate analysis showed that nebulized SABA, age, GOLD grades, antibiotics use, comorbidities, systemic corticosteroid, and inpatient visits in the past year were associated with hospital expenditure, while nebulized SABA, age, inpatient visit in the past year, comorbidities, and antibiotics use were associated with LOS. The risk of hospital expenditure in the multivariable model revealed the increasing odds of expenditure with albuterol, older age, and worse obstruction severity, while nebulized SABA, older age, and inpatient visits in the past year were identified as the risk factors for LOS.

**Table 3:** Logistic regression of the risk factors associated with hospital costs in COPD patients Backward selection with an alpha level of removal of 0.05 was used. The following variables were removed from the model: antibiotics use, comorbidities, systemic corticosteroids, and inpatient visits in the past year. CI: confidence interval; GOLD: Global Initiative for Chronic Obstructive Lung Disease; SAMA: short-acting muscarinic agonist; SC: systemic corticosteroid

	Univariate		Multivariate	
	Odds ratio (95% CI)	Odds ratio P value	Odds ratio (95% CI)	OR P value
Albuterol	2.16 (1.14-4.07)	0.018	2.27 (1.12-4.61)	0.024
Age, years	-	-	-	-
40-60	1	-	-	-
60-80	3.58 (1.34-9.56)	0.011	4.26 (1.50-12.08)	0.006
80-100	11.67 (2.77-49.13)	< .001>	17.34 (3.71-81.12)	< .001>
GOLD grade	-	-	-	-
GOLD 1	1	-	-	-
GOLD 2	1.67 (0.39-7.12)	0.490	1.14 (0.24-5.37)	0.870
GOLD 3	4.93 (1.23-19.74)	0.024	3.50 (0.80-15.34)	0.096
GOLD 4	4.81 (1.16-19.99)	0.030	4.30 (0.94-19.66)	0.060
Number of comorbidities	-	-	-	-
0	1	-	-	-
1	2.04 (1.01-4.13)	0.047	-	-
2	2.50 (0.95-6.60)	0.064	-	-
Outpatient visits >1 in the past year	1.14 (0.56-2.34)	0.715	-	-
Inpatient visits >1 in the past year	-	-	-	-
Inpatient SAMA use	2.05 (0.37-11.54)	0.414	-	-
Inpatient SC use	2.35 (1.23-4.51)	0.010	-	-
Inpatient use of antibiotics types	-	-	-	-
0	1	-	-	-
1	5.58 (1.19-26.19)	0.029	-	-
≥2	16.49 (2.51-108.55)	0.004	-	-

**Table 4:** Logistic regression of the risk factors associated with LOS in COPD patients Backward selection with an alpha level of removal of 0.05 was used. The following variables were removed from the model: comorbidities and antibiotic use. CI: confidence interval; GOLD: Global Initiative for Chronic Obstructive Lung Disease; SAMA: short-acting muscarinic agonist; SC: systemic corticosteroid

	Univariate		Multivariate	
	Odds ratio (95% CI)	Odds ratio P value	Odds ratio (95% CI)	OR P value
Albuterol	2.16 (1.14-4.08)	0.018	2.48 (1.27-4.87)	0.008
Age, years	-	-	-	-
40-60	1	-	-	-
60-80	2.62 (1.02-6.72)	0.045	2.38 (0.90-6.29)	0.081
80-100	5.43 (1.47-20.08)	0.011	5.69 (1.46-22.26)	0.012
GOLD grade	-	-	-	-
GOLD 1	1	-	-	-
GOLD 2	1.26 (0.33-4.85)	0.737	-	-
GOLD 3	2.25 (0.63-8.08)	0.214	-	-
GOLD 4	3.25 (0.87-12.19)	0.081	-	-
Number of comorbidities	-	-	-	-
0	1	-	-	-
1	1.56 (0.77-3.14)	0.213	-	-
2	3.21 (1.19-8.71)	0.022	-	-
Outpatient visits >1 in the past year	1.31 (0.63-2.70)	0.467	-	-
Inpatient visits >1 in the past year	2.13 (1.12-4.04)	0.021	2.09 (1.06-4.15)	0.034
Inpatient SAMA use	2.28 (0.41-12.83)	0.349	-	-
Inpatient SC use	1.16 (0.61-2.19)	0.655	-	-
Inpatient use of antibiotics types	-	-	-	-
0	1	-	-	-
1	1.96 (0.57-6.67)	0.284	-	-
≥2	4.95 (1.02-24.08)	0.048	-	-

## Discussion

AECOPD is a primary cause of hospitalization, morbidity, and mortality in COPD patients, and it also increases public health expenditure [17]. Ren et al. reported that the 30-day all-cause mortality rate ranged from 0.00 to 2.23/1,000 person-months (pm), and the readmission rate was between 30.64 and 33.52/1,000 pm [2].

In this retrospective cohort study of hospitalized COPD patients, levalbuterol was associated with reduced hospital stay and lower hospital expenditure than albuterol treatment. Additionally, receiving albuterol was identified as an independent risk factor for a longer hospital stay and higher hospital expenditure.

Our results in costs and LOS are comparable to two earlier retrospective studies. Truitt et al., in a retrospective chart review study with 177 COPD patients, showed that levalbuterol treatment resulted in a shorter LOS (albuterol vs. levalbuterol, 5.1 vs. 6.1 days, P = 0.07), with $554 lower hospital costs (albuterol vs. levalbuterol, $3,506 vs. $2,952, P = 0.0013) [10]. Additionally, a database study, which obtained 10,958 patients’ data from the Medicaid database, reported a noteworthy cost savings of about $967 in COPD patients who used levalbuterol (P = 0.0031) compared to those with albuterol [18]. However, note that levalbuterol’s benefits did not reach a consensus. A randomized, prospective, open-label study of data from 486 patients found no significant difference in LOS (albuterol vs. levalbuterol, 65.7 and 70.6 hours, P = nonsignificant) or total hospital costs (albuterol vs. levalbuterol, $4,899.41 and $4,869.30, P = 0.94) between treatment groups receiving levalbuterol and racemic albuterol [11]. However, this study did not separate individual asthma and COPD subpopulations that may deviate from the outcomes because of the different responses to beta-agonists among asthma and COPD patients. Moreover, a single-center, randomized, prospective, open-label study with 112 patients demonstrated a significantly greater LOS (albuterol vs. levalbuterol, 6.8 vs. 8.5 days, P = 0.04) and higher total treatment costs in the levalbuterol group (albuterol vs. levalbuterol, $5,772 vs. $8,003, P = 0.0006) [12]. Asthma and COPD patients had not been separated in this study either, and there is a disease grade absence in baseline information (e.g., patients’ hospitalization or emergency visit in the past year, and lung function was not reported in baseline), which may lead to the unequal disease severity in the two treatments.

This study is also in line with a cost-utility analysis, which compared nebulized levalbuterol with albuterol in hospitalized COPD patients [9]. In that cost-utility model, Chen et al. [9] considered both direct and indirect medical costs, and their results show a ¥495.7 ($105.1) savings per hospitalization per patient. The difference in a scenario analysis that did not consider indirect costs is ¥263.6 ($55.9). In our study, the difference is ¥1,422.4 ($301.6) per hospitalization per patient. The gap may be because of the various cost components. However, both of our studies support the cost-saving benefits of levalbuterol. This is the first study to validate the benefits of levalbuterol in the Chinese population. The results of our study may be valuable in guiding future intervention research focused on optimizing medication use in individuals with COPD. Moreover, our study has good potential for improving the precision of the prediction of the cost-utility model.

The implications of our findings should be interpreted in light of the study’s limitations. First, the generalizability of the results may be restricted because of the limited sample size and geographic scope. Our data were derived from three hospitals in Chongqing, which may not represent other areas in China and may not accurately reflect the condition in community settings. Second, as our study was retrospective, missing data became a potential pitfall, and there might be other risk factors for the LOS and hospital costs. Moreover, our data were extracted from a medical database, which was created for clinical use, not research. Hence, despite the quality concerns associated with routinely collected data, hospitalized patients are subjected to close monitoring, which could enhance the accuracy of the data.

## Conclusions

In this study, we reviewed the COPD cases at three hospitals in China. Patients who received levalbuterol demonstrated a reduced hospital stay and lower hospital expenditures. Compared with levalbuterol, albuterol treatment was an independent risk factor for a longer hospital stay and higher hospital expenditures.

Additionally, future studies could analyze these two subsets of patients after the withdrawal of SABAs and the impact of clinical deterioration requiring higher levels of care and assess adverse drug effects in multiple centers to improve the applicability of bronchodilator stewardship in this subset of patients.
